# Vaccines and the Eye: Current Understanding of the Molecular and Immunological Effects of Vaccination on the Eye

**DOI:** 10.3390/ijms25094755

**Published:** 2024-04-26

**Authors:** Yaru Zou, Koju Kamoi, Yuan Zong, Jing Zhang, Mingming Yang, Kyoko Ohno-Matsui

**Affiliations:** Department of Ophthalmology and Visual Science, Graduate School of Medical and Dental Sciences, Tokyo Medical and Dental University, Tokyo 113-8510, Japan; alicezouyaru519@gmail.com (Y.Z.); zongyuan666.oph@tmd.ac.jp (Y.Z.); zhangjing.oph@tmd.ac.jp (J.Z.); yangmm12.oph@tmd.ac.jp (M.Y.); k.ohno.oph@tmd.ac.jp (K.O.-M.)

**Keywords:** vaccines, ocular inflammation, molecular, immunomodulation, molecular mimicry, COVID-19

## Abstract

Vaccination is a public health cornerstone that protects against numerous infectious diseases. Despite its benefits, immunization implications on ocular health warrant thorough investigation, particularly in the context of vaccine-induced ocular inflammation. This review aimed to elucidate the complex interplay between vaccination and the eye, focusing on the molecular and immunological pathways implicated in vaccine-associated ocular adverse effects. Through an in-depth analysis of recent advancements and the existing literature, we explored various mechanisms of vaccine-induced ocular inflammation, such as direct infection by live attenuated vaccines, immune complex formation, adjuvant-induced autoimmunity, molecular mimicry, hypersensitivity reactions, PEG-induced allergic reactions, Type 1 IFN activation, free extracellular RNA, and specific components. We further examined the specific ocular conditions associated with vaccination, such as uveitis, optic neuritis, and retinitis, and discussed the potential impact of novel vaccines, including those against SARS-CoV-2. This review sheds light on the intricate relationships between vaccination, the immune system, and ocular tissues, offering insights into informed discussions and future research directions aimed at optimizing vaccine safety and ophthalmological care. Our analysis underscores the importance of vigilance and further research to understand and mitigate the ocular side effects of vaccines, thereby ensuring the continued success of vaccination programs, while preserving ocular health.

## 1. Introduction

At the end of 2019, the global outbreak of the coronavirus disease 2019 (COVID-19) caused by severe acute respiratory syndrome coronavirus 2 (SARS-CoV-2) posed an unprecedented challenge worldwide. With various COVID-19 vaccines proving effective in clinical trials, the efficacy and safety of such vaccines are weighing on the minds of every citizen. The development and testing of vaccines are currently hot topics in the fields of public health, basic research, and clinical studies [1]. Despite the numerous benefits of vaccines, excessive stimulation of the immune system may become more apparent after repeated vaccinations or adjuvant use [2]. Given the large number of people vaccinated annually, there is an urgent need to closely monitor and explore adverse reactions to vaccines. Some scholars believe that research on vaccine administration and autoimmune diseases is coincidental, and excessive exploration could create “false myths”, leading to vaccine hesitancy and a reduction in vaccination coverage [3]. However, it must be acknowledged that a connection exists between the two, albeit rarely [4]. The purpose of investigating these mechanisms is to improve public health and promote vaccine optimization. Therefore, the open debate on adverse reactions to vaccines should not be viewed as anti-vaccine activism [5].

In recent years, independent clinical practitioners from various parts of the world have begun to document adverse reactions, encompassing both systemic and localized symptoms, following vaccine administration. As early as 2013, Salmon et al. published a large-scale population study in the Lancet, which revealed a significant association between influenza vaccine administration and Guillain–Barré syndrome [6]. Simultaneously, a plethora of research on vaccine-related side effects emerged. Some studies have found a close correlation between the administration of the poliovirus vaccine and neurological complications [7], while others have explored the association between measles–mumps–rubella (MMR) vaccine administration and immune thrombocytopenic purpura [8].

As a vital organ of the human body, the eyes have consistently garnered the attention of medical professionals, and vaccine-related ocular side effects have continually been reported. Research has indicated that uveitis is the most common ocular complication following hepatitis B vaccine administration [9], and studies have shown that the annual incidence of postvaccination uveitis ranges from 8 to 13 cases per 100,000 cases/year [10]. A recent retrospective review of ocular adverse events induced by vaccines between 2010 and 2020 emphasized various vaccine-related ocular reactions, including optic neuritis, uveitis, and retinitis [11]. Our previous study summarized 61 cases of post-antiviral vaccination uveitis over a 40-year period [12]. In most cases of these ocular inflammations, they are self-limiting or recover after treatment with systematic steroids. However, there remains a subset of patients who, despite receiving steroid treatment and long-term follow-up, do not improve. They may experience permanent visual impairment, visual field defects, and require prolonged steroid therapy, which may ultimately develop into lifelong permanent complications. Therefore, exploring the mechanisms behind it is crucial to reduce the occurrence of this situation. Nowadays, numerous speculations exist regarding the immunological and molecular mechanisms underlying vaccine-related ocular inflammation. This review summarizes some mainstream mechanisms of vaccine-related ocular inflammation and provides insights and directions for future foundational research in this field.

## 2. Methods of Literature Search

The PubMed, Embase, and Cochrane Library databases (from 1983 to June 2023) were used to search the relevant publications that included case reports and series, as mentioned in previous studies [12]. Cited cases featuring relevant mechanisms are included as examples.

## 3. Molecular and Immunological Effects

### 3.1. Direct Inflammation Due to Live Attenuated Vaccines

The first mechanism involves direct infection by viruses from live but attenuated vaccines such as the VZV Oka strain in Zostavax by Merck [13]. A study on adverse events after vaccination revealed that, 42 days post-immunization, adverse events related to the herpes zoster vaccine (of any other type) were significantly more common in vaccinated people than in placebo recipients (*p* < 0.05) [14]. In 2017, Grillo et al. conducted a retrospective study on cases of VZV-related keratitis and identified 24 patients, including 15 children and 9 adults, with this eye inflammation problem [15]. Though all patients experienced unilateral symptoms, in most cases, these symptoms improved significantly with the use of corticosteroids and antiviral drugs.

Our previous retrospective systematic review encompassed 12 cases (among 16 patients) of VZV-related ocular inflammation [12], with most patients aged 60 years or older having a history of varying degrees of immunosuppression and metabolic disorders. Importantly, almost all patients tested positive for VZV DNA, and some even tested positive for the Oka strain of the VZV vaccine [16,17], strongly suggesting a close association between ocular inflammation and administration of a still-active VZV vaccine.

Other attenuated live vaccines include the MMR vaccine produced by Merck (Kenilworth, NJ, USA), the nasal spray influenza vaccine FluMist produced by MedImmune/AstraZeneca (Gaithersburg, MD, USA), the rotavirus vaccines RotaTeq and Rotarix produced by Merck and GlaxoSmithKline (Brentford, UK), respectively, and the yellow fever vaccine YF-Vax produced by Sanofi (Paris, France). One patient, who received the MMR vaccine at nine months of age, developed unilateral anterior uveitis with iris abnormalities and cataracts three months later. A second injection was administered when ocular symptoms were present. Laboratory tests revealed HLA-B51 positivity. Through aqueous humor analysis, researchers found a higher concentration of rubella-specific immunoglobulin G in the affected eye than in the unaffected eye. This underscores the potentially significant role of the attenuated MMR vaccine in ocular inflammation [18].

Multiple evanescent white dot syndrome (MEWDS) is a rare condition characterized by the presence of multiple small white or yellow lesions in the outer retina and retinal pigment epithelium, typically unilaterally, with an orange appearance in the central macula [19]. MEWDS cases have been described following vaccinations against hepatitis A, yellow fever, and influenza [20,21,22]. Up to half of patients experience viral prodromal symptoms, and increases in serum immunoglobulin IgG and IgM levels during the acute phase of MEWDS suggest a possible association with direct infection from attenuated live vaccines.

### 3.2. Autoimmune/Inflammatory Syndrome Induced by Adjuvants (ASIA)

The second mechanism entails inflammation triggering through one or more adjuvants, usually aluminum salts. These adjuvants are frequently employed in inactivated vaccines or subunit/conjugate vaccines that include targeted pathogenic components or fragments, such as those found in HPV, HBV, and COVID-19 vaccines [23]. ASIA and autoimmune disorders are collectively known as the Shoenfeld’s syndrome [24,25]. This syndrome manifests more frequently in individuals with a familial or personal history of autoimmune diseases. Furthermore, Watad et al. found that, in patients with clearly defined immune disorders, exposure to adjuvants was more frequently followed by the development of polygenic autoimmune diseases than of polygenic inflammatory diseases (92.7% vs. 5.8%, *p* < 0.001), suggesting a genetic predisposition [1]. Additionally, according to a survey conducted by the ASIA International Registry Center on 300 individuals with adjuvant-induced autoimmune inflammatory spectrum, the majority of patients were females with a median age of 38 years [25]. This gender predominance has been confirmed in subsequent studies, with females comprising as much as 89% of the affected population [1]. Common systemic symptoms include chronic fatigue, joint pain, muscle aches, and fever, as well as conditions such as Sjögren’s syndrome and cognitive neurological disorders.

#### 3.2.1. Inflammation Caused by Adjuvant and Adaptive Immune Component Interactions

Adjuvants can engage with various elements of the immune system, encompassing both innate and adaptive immunity [26,27]. To facilitate the comprehension of the intricate interplay between vaccine adjuvants and immune-mediated events, Koenig et al. proposed the following classification based on the immune elements involved [28]: (1) Pure innate diseases resulting from inherent immune cell dysregulation, compromised immune barriers, excessive tissue repair, and remodeling processes [29], and (2) adaptive immune diseases, including innate immune disorders that are characterized by an adjuvant-driven autoinflammatory phenotype (due to the dysregulation of B and T cells and the activation of innate immune components, such as neutrophils, macrophages, and inflammatory mediators) and common autoimmune disorders (due to the activation of adaptive immunity, involving antigen-presenting cells and T and B cells that produce antibodies in response to adjuvants). Common autoimmune disorders include ocular vasculitis, optic neuritis, neuromyelitis optica, Behçet’s disease, Vogt–Koyanagi–Harada (VKH) syndrome, and sarcoidosis. Immune cells in the eyes are primarily concentrated in the conjunctiva and cornea.

**Figure 1:**
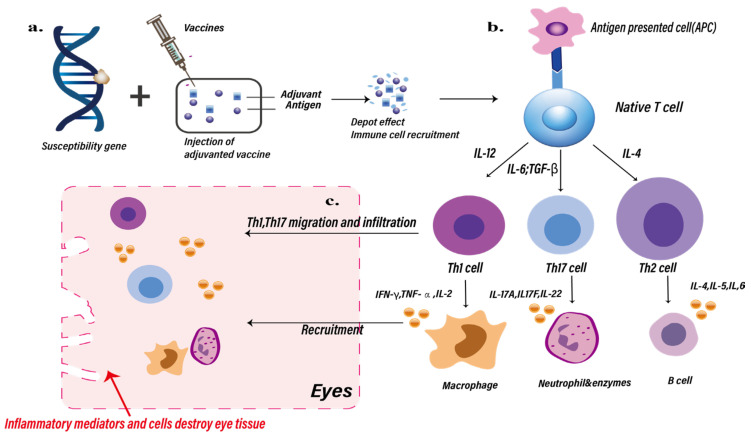
Potential pathways/mechanisms of ocular inflammation induced by vaccine adjuvants. (**a**) After patients carrying genetic susceptibility genes are injected with vaccines containing adjuvants, the recruitment depot effect of immune cells will be promoted, thereby promoting the activation of more antigen-presenting cells (APCs). (**b**) The interaction between APCs and T cells promotes the production of greater numbers and types of helper T cell, cytokines, inflammatory cells, and B cells. (**c**) Among them, Th1 and Th17 cells migrate and infiltrate into eye tissue, and macrophages and neutrophils can be recruited into the eye and cause damage to eye tissues.

Figure 1 shows a schematic representation of the potential mechanisms involved in postvaccination ocular inflammation, based on Watad et al. [1].

In 2019, Sood et al. reported a case of ‘recurrent postvaccination uveitis’, in which a patient experienced loss of vision in the right eye after receiving the first dose of the hepatitis B vaccine. The initial diagnosis was noninfectious panuveitis with choroiditis, and subsequent occurrences led to increasing deterioration of the eye [24]. This kind of non-infectious inflammation is most likely due to the adjuvants (such as aluminum) contained in the hepatitis B vaccine, which enhance immunogenic activity through a combination of mechanisms, including the sustained release of cytokines, chemokines, and antigens (depot effect), activation of antigen-presenting cells, and antibody production. Similarly, Fraunfelder et al. documented the development of uveitis in 32 patients 3 days after receiving an aluminum-adjuvanted hepatitis B vaccine. Among them, 15 patients experienced inflammation after the first dose, 3 after the second dose, and 3 more after the third dose. Notably, two patients experienced recurrent uveitis upon revaccination, strongly suggesting a potential causal relationship between the vaccine and uveitis [30] (Figure 1 and Table 1).

#### 3.2.2. Aluminum Particles That Delay Vaccine Dissolution Are Captured by Immune Cells

In 2019, Gherardi et al. published a study on aluminum and myalgic encephalomyelitis/chronic fatigue syndrome [52]. Using epidemiological, clinical, and experimental evidence, the authors demonstrated that myalgic encephalomyelitis/chronic fatigue syndrome is a major adverse reaction to vaccines. The primary mechanism is as follows: vaccines containing aluminum adjuvants, especially those with poorly degradable particle formulations, do not rapidly dissolve in the extracellular space, but accumulate at the injection site, forming aluminum clusters. This pathology has been experimentally replicated in animals [53,54]. In contrast to the rapid elimination of soluble aluminum through intravenous injection [55], intramuscular injection of isotope-labeled aluminum hydroxide results in a significantly slower excretion rate in urine, representing, in rabbits, 6% of the injected dose after 28 days [56]. In monkeys, aluminum adjuvant-induced macrophagic myofasciitis develops after administration of the diphtheria, tetanus, pertussis (DTP) vaccine, in doses equivalent to 14–21 times the human dose, and it takes up to 6 months to completely clear this adjuvant from the injected muscle [53]. Similarly, most humans appear to clear the adjuvant from the injected muscle within a few months [52].

This delayed dissolution causes immune system cells to quickly capture the injected aluminum particles and transport them to various organs, including the brain, interfering with the tricarboxylic acid cycle (TAC) in brain cells and causing metabolic and chronic neurotoxic effects. Although this mechanism is often considered irrelevant to humans, reports indicate that sheep (a large animal similar to humans) [57] can develop neurological diseases, including encephalomyelitis with behavioral changes and subsequent spinal cord neurodegenerative lesions, after multiple vaccinations with aluminum-containing adjuvants [58]. While there are currently no reported cases of ocular side effects associated with this mechanism, it is highly plausible that aluminum particles captured by immune cells can lead to ocular neurodegeneration through the blood–eye barrier. This hypothesis lays the foundation for subsequent research on adjuvant retention-related ocular diseases.

#### 3.2.3. Aluminum-Related Dorsal Root Ganglion Injury

The presence of aluminum hydroxide or aluminum phosphate salts in HPV vaccines is known to enhance their immunogenicity, prompting a more effective production of antibodies and immune memory. However, cases of neuro-optic inflammation following HPV vaccination have also been reported. One case involved a 30-year-old female who experienced visual impairment of the left eye, abnormal eye movement, eye tenderness, and color blindness after receiving the first dose of the HPV vaccine. While there was a slight improvement with corticosteroid use, the patient developed recurrent visual impairment and eye movement pain in the right eye three days after the second dose, with magnetic resonance imaging (MRI) revealing enhanced contrast of the right optic nerve, while infectious markers and cerebrospinal fluid levels were normal. Recurrent optic neuritis following vaccination strongly suggests an association with this vaccine [33]. Moreover, Menge et al. reported a series of four cases of neuromyelitis optica following HPV vaccination [32].

Some researchers have proposed that this mechanism may be related to the dorsal root ganglia (DRG). The DRG is a crucial neural site, where various external substances, including viruses, immune complexes, and vaccines, can induce neuropathic pain and autonomic nerve disorders. Recent studies in mice have suggested that, after vaccination, sensory neurons in the DRG can isolate and retain antigen-specific antibodies released by plasma cells [59]. Captured vaccine-induced antigen-specific antibodies can theoretically cross-react with DRG epitopes, inducing neuropathic pain and autonomic nerve disorders in susceptible individuals. Another animal model suggested that aluminum adjuvants may impair the DRG [60].

#### 3.2.4. AS01B-Induced Ocular Inflammation

The first live attenuated vaccine for herpes zoster, Zostavax (Merck), was approved by the U.S. Food and Drug Administration (FDA) in 2006 [16]. However, there were reports of patients experiencing reactivation of herpes zoster ophthalmicus (HZO), including the development of keratitis, corneal perforation, and acute retinal necrosis, following Zostavax administration [61,62]. Subsequently, the recombinant subunit vaccine RZV (Shingrix; GlaxoSmithKline, Research Triangle Park, NC, USA) was introduced. In clinical trials evaluated by the FDA, Shingrix demonstrated an efficacy of up to 97% in reducing the incidence of herpes zoster in individuals aged 50 and above [63].

However, cases of vaccine-related ocular inflammation associated with RZV administration have also been reported. Jabbour et al. reported the case of a 78-year-old woman with a history of HZO who experienced pain and photophobia one week after receiving the RZV vaccine [35]. Slit-lamp examination revealed necrotizing stromal keratitis and diffuse corneal edema. Another case involved a 53-year-old woman who developed uveitis four days after vaccination [34]. RZV-induced ocular inflammation may be related to the novel adjuvant AS01B present in the vaccine [64].

AS01 is an adjuvant system contains 3-O-desacyl-4′-monophosphoryl lipid A (MPL), the saponin derived QS21, dioleoylphosphatidylcholine (a phospholipid), and cholesterol [64]. MPL is a modified form of lipopolysaccharide (LPS), primarily derived from that of *Salmonella minnesota*. It activates innate immunity by stimulating antigen-presenting cells expressing Toll-like Receptor 4. Notably, previous studies have used LPS as a mediator to induce the occurrence of uveitis [65]. QS21 stimulates the innate immune response monocyte pathway through a specific mechanism [66], and its lipid formulation enhances antigen presentation [67]. Immunization with AS01 adjuvant, which contains low-dose MPL and QS21, induces a higher CD4+ T-cell and humoral immune response than immunization with glycoprotein E without adjuvant or glycoprotein E + AS01 (containing lower doses of MPL and QS21 than AS01B) [68,69]. So far, there is no basic research to confirm this hypothesis. Therefore, the safety of this novel adjuvant, AS01B, requires further investigation.

### 3.3. Type III Hypersensitivity/Immune Complex Disease and Type IV Hypersensitivity/Delayed-Type Hypersensitivity (DTH)

Vaccine-related neurological disorders, such as optic neuritis, neuromyelitis optica spectrum disorders (NMOSD), and Guillain–Barré syndrome, were reported as early as 1973 [70]. Previously, researchers believed that this immune response primarily focused on the cellular immune system attacking the myelin sheath, leading to subsequent demyelination [71,72]. These explanations were largely based on the high clinical and histopathological similarities between experimental allergic neuritis (EAN) and experimental allergic encephalomyelitis (EAE) [73]. Both diseases appeared to be triggered by delayed hypersensitivity reactions (type IV hypersensitivity), and hypotheses were supported by evidence of sensitized lymphocytes causing demyelination in some neurological disorders, such as Guillain–Barré syndrome and Bell’s palsy [74].

**Figure 2:**
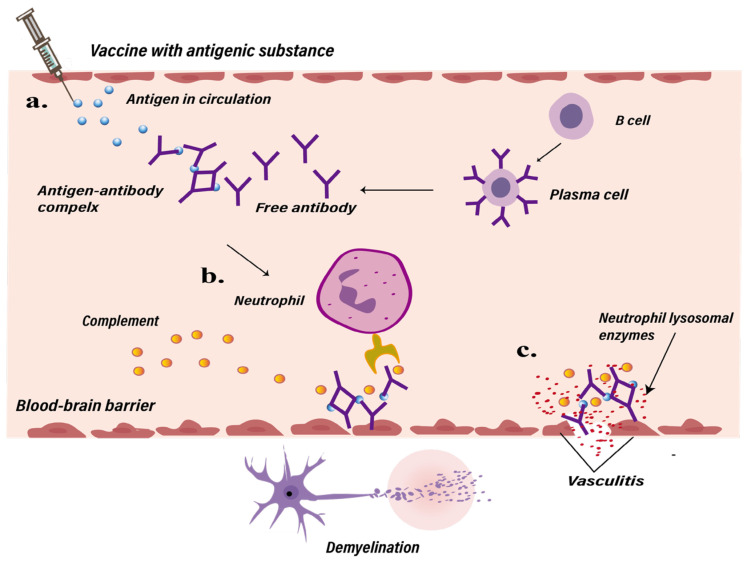
Possible pathways to post-vaccination demyelinating disease mediated by hypersensitivity reactions. (**a**) After vaccination with antigenic substances, antigen-antibody immune complexes are synthesized in the bloodstream, triggering activation of the complement system and inflammatory cells such as neutrophils. (**b**) Neutrophils release inflammatory mediators, lysosomal enzymes, and complement, which increase vascular permeability, leading to tissue damage and vascular inflammation. (**c**) This process can compromise the blood-brain barrier, potentially resulting in demyelinating diseases.

However, not all patients exhibited sensitized lymphocytes, and some cases pointed out that immunosuppressed patients may also develop Guillain–Barré syndrome [75]. In 1979, Poser et al. found that patients with neurological infections often exhibited the histopathological characteristics of vasculitis accompanied by demyelination [73]. Subsequent studies detected circulating antigen–antibody complexes in the serum of patients with post-infectious diseases, proposing an alternative hypothesis to explain primary vascular lesions and secondary demyelination [76,77]. Certain substances in vaccines possess antigenic properties, leading to the formation of immune complexes through interactions between antigens and antibodies, triggering a type III hypersensitivity reaction in which deposited immune complexes activate the complement system and neutrophils. The activation of the complement system induces inflammation, leading to tissue damage, the recruitment of neutrophils and monocytes, and increased vascular permeability. Neutrophils release lysosomal enzymes that cause tissue injury and vasculitis. Ultimately, this causes damage to the blood-brain barrier, leading to demyelinating diseases [40], similar to the presumed immune complex-mediated vasculitis that occurs in some patients infected with HBV [78] (Figure 2 and Table 1).

This mechanism has been confirmed in subsequent reports on vaccine-related optic neuritis. Gupta et al. reported in 2005 the case of a patient who received an anti-rabies vaccine prepared from sheep brain tissue [43]. On the 11th day postvaccination, the patient developed headache accompanied by eye movement pain. Fundus fluorescein angiography revealed retinal vascular tortuosity with disc leakage, and axial T2-weighted MRI scans showed bilateral optic nerve signal enhancement, which was consistent with the diagnosis of bilateral optic neuritis. The authors suggested that the antigenicity of sheep brain tissue allowed lymphocytes to synthesize specific viral antibodies to enter the brain, leading to inflammation and demyelination. With the introduction of anti-rabies vaccines made from chicken embryos, the corresponding adverse effects have been reported. Saxena et al. reported the case of a 56-year-old patient who experienced acute painless vision loss in the right eye after receiving the chick embryo cell anti-rabies vaccine (Rabipur; Hoechst Marion Roussel) [39]. Fundus examination revealed optic disc congestion and disc edema, while MRI showed optic nerve signal enhancement, diagnosed as optic neuritis. Given a previous case of Guillain–Barré syndrome following the use of a purified chicken embryo cell anti-rabies vaccine [79], it was suspected that these alterations in the eye might be attributable to the chicken embryo antigen.

The mechanisms underlying the hypersensitivity reactions extend beyond neurological damage, as confirmed in other reported cases of vaccine-related ocular adverse reactions. One patient, for example, developed uveitis within a typical interval of 3–4 days post-antigen exposure after receiving the purified HBV vaccine, supporting an immune complex-mediated disease [41]. Additionally, the results of lymphocyte stimulation tests also raised the suspicion of a cell-mediated immune response. Furthermore, Yang et al. reported a patient who developed MEWDS after receiving an embryonated egg anti-rabies vaccine [44]. Visual field examination revealed enlargement of the blind spots, and fundus fluorescein angiography indicated choroidal background hyperfluorescence with diffuse fluorescein leakage. Considering that MEWDS is generally believed to be associated with immune mechanisms, and that the patient showed significant relief after steroid therapy, the occurrence of MEWDS may be explained by autoimmune-mediated inflammatory mechanisms.

### 3.4. Molecular Mimicry

Molecular mimicry is an autoimmune reaction triggered when a host is exposed to antigens that share amino acid homology with the amino acid chains of the organs of the host, which may lead to a host’s immune system response against these organs [80]. Molecular mimicry is an important mechanism in the acquisition of autoimmunity and this hypothesis has been proven in animal experiments [81,82]. Westall and Root-Bernstein hypothesized that the basis for the occurrence of acquired autoimmune responses lies in the presence of dual antigens under the influence of immunological adjuvants [83]. They proposed the following additional requirements for the occurrence of acquired autoimmunity: (1) One antigen must exhibit molecular mimicry with human tissues, while two other antigens must demonstrate chemical complementarity, and (2) immunological adjuvants should be present. The resulting syndrome was named multiple antigen-mediated autoimmunity (MAMA).

Foundational research on uveitis, experimentally induced through molecular simulation of immune mechanisms, began quite early [84,85,86,87,88]. Researchers initiated the immune system by subcutaneous injection of bacterial products, such as LPS and lipoteichoic acid (LTA), which are unrelated to the retinal or uveal tissues. Activation involved the retinal S antigen and interphotoreceptor retinoid binding protein (IRBP), which triggered T cells and caused inflammation of the uvea. Both bacterial and viral products were able to induce inflammation. Furthermore, during the immune response, viral antigens or cultured viral products may initiate early events in the immune activation pathway via antigen mimicry, thereby inducing uveitis [31].

**Figure 3:**
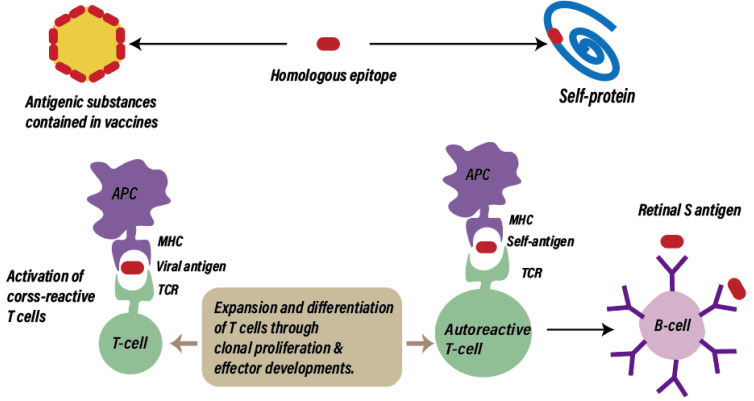
Possible pathways leading to postvaccination ocular disease by molecular mimicry. When antigenic substance contained in vaccine and a self-protein contain peptide epitope recognized by the same T cell, exogenous antigen-specific T cells can attack not only infected cells but also uninfected cells displaying the same epitope. MHC, Major Histocompatibility Complex; TCR, T-cell receptor.

Computer-assisted analyses have indicated that the HPV 16 type E7 oncoprotein exhibits extensive similarity to several human proteins involved in key regulatory processes, and different E7 peptide sequences are present in the same human proteins [89]. While the shared motifs between viral proteins and normal cellular molecules could contribute to a lack of immunogenicity in HPV infections, mimicry between HPV4 and human proteins could cause uveitis or other autoimmune reactions induced by HPV4. Additionally, it has been confirmed that molecular mimicry and antigenic similarity between *Mycobacterium tuberculosis* proteins and retinal antigens are potentially responsible for the uveitis caused by Bacillus Calmette–Guérin (BCG) vaccination [90]. Chen et al. described the case of a 27-year-old woman who developed acute panuveitis rapidly (4 days) after receiving the third dose of the quadrivalent HPV vaccine; analysis of skin samples of the patient showed intimal hyperplasia and vessel wall hyalinization accompanied by the infiltration of a large number of monocytes, macrophages, and lymphocytes, consistent with the vasculitis diagnosis, and suggesting that HPV vaccination might be related to immune-reactive vasculitis [45]. In addition, the cases of choroiditis described by Khalifa et al. lacked viral prodromal symptoms, thus favoring molecular mimicry rather than nonspecific immune responses when considering the underlying mechanisms [46] (Figure 3 and Table 1).

#### 3.4.1. Acute Posterior Multifocal Lamellar Pigment (APMPPE) and Molecular Mimicry

APMPPE is a self-limiting syndrome-driven uveitis classified as a heterogeneous white spot syndrome [91]. VZV, HAV, HBV, *Neisseria meningitidis*, yellow fever, typhoid fever, and influenza vaccines appear to be associated with the occurrence of APMPPE [47,92,93]. Some studies have suggested that APMPPE is caused by ischemic damage to the choriocapillaris and retinal pigment epithelium due to obstructive vasculitis. The triggers include immunization and congenital autoimmune diseases. Some studies on the MHC (HLA) class I (MHC-I) and class II (MHC-II) cell surface antigen receptors responsible for immune regulation in patients with APMPPE have pointed out that those patients in which MHC-I HLA-B7 and MHC-II HLA-DR2 antigens were present had relative risks of 3.38 and 3.34 of developing APMPPE, respectively [94]. MHC-I and MHC-II are antigenic peptides that bind to CD4+ and CD8+ T cells, respectively, to mediate immune responses. MHC-I class molecules are widely present in nucleated cells, including the vascular endothelial cells of the iris, retina, ciliary body, and choroid. The MHC-II class molecule distribution is more restricted; they are mainly found in the cells of the limbus and uvea, including the choroid, of the eye. When exposed to foreign infections, specific HLA genotypes present exogenous peptide fragments to T cells to induce antibodies. This exogenous fragment may cross-react with a homologous self-antigen peptide present in the related HLA gene product, producing autoantibodies. This immune response hypothesis, a hypothesis based on molecular mimicry and cross-reactivity, is similar to that for HLA-B27-positive uveitis and ankylosing spondylitis [95]. Notably, several HLA receptor subtypes have been identified in some reports of APMPPE cases, including HLA-DR2, HLA-B7, HLA-B27, HLA-A3, and HLA-C7 [47,96,97]. Therefore, it is likely that these receptors act synergistically with live infectious vaccine epitopes to induce this chorioretinopathy.

#### 3.4.2. Tubulointerstitial Nephritis and Uveitis (TINU) Syndrome and Molecular Mimicry

Sawai et al. reported the cases of two patients who developed TINU following vaccination with the HPV vaccine [42]. Both patients, who were previously in good health, exhibited slight elevations in serum creatinine, hemoglobin, C-reactive protein, and erythrocyte sedimentation rate, and corticosteroid therapy proved effective. TINU is an autoimmune disease that is mediated by both cellular and humoral responses [98]. Human leukocyte antigen class II (HLA class II) molecules, such as HLA-DRB1*0102, play a role in presenting exogenous antigens to CD4+ helper T cells during the early stages of cellular immunity. Specific subtypes within HLA class II may influence the immune response, rendering individuals sensitive to otherwise harmless antigens [99]. The involvement of both the kidney tubules and uvea in the disease may be explained by the presence of shared or similar antigens in these organs [100]. The significance of T-cell-mediated immunity is supported by kidney histology, demonstrating tubulointerstitial infiltrates primarily composed of helper/inducer T-cell subsets [101,102]. FOXP3+ T regulatory lymphocytes (T-regs) are crucial for maintaining self-tolerance and tissue homeostasis, and aberrant T-reg function has been implicated in various autoimmune diseases and malignancies [103]. T-reg cells were identified in kidney biopsies from pediatric patients with TINU. Interestingly, in those cases of TINU with chronic uveitis, the density of T-regs was lower, suggesting that an autoimmune mechanism was contributing to a persistent inflammatory response [104].

In addition, the presence of autoantibodies against modified C-reactive protein (mCRP) in the kidney and eye tissues suggests the involvement of humoral immunity [105]. It was determined that the prevalence of serum anti-mCRP autoantibodies was significantly higher in patients with TINU syndrome than in those with other renal autoimmune diseases or healthy controls, suggesting a possible disease-specific association [106]. Furthermore, in patients with acute interstitial nephritis, elevated anti-mCRP antibodies may predict the subsequent development of uveitis [107]. This is consistent with clinical evidence, as the two patients reported by Sawai et al. had interstitial nephritis and subsequently developed uveitis [42]. Therefore, the kidneys may be the primary target of a sequential process that triggers an inflammatory cascade with secondary effects on the eye. The etiology of TINU may be associated with drug use and infections. Moreover, studies have reported associations with VZV vaccines, nonsteroidal anti-inflammatory drugs, and antibiotics [108,109]. Although cases of vaccine-related TINU are relatively rare, based on the aforementioned findings, vaccine-induced hypersensitivity reactions are likely contributors to this condition.

### 3.5. PEG-Induced Allergic Reactions

Coronaviruses are a group of single-stranded positive-sense RNA viruses that mainly bind to the angiotensin-converting enzyme 2 (ACE2) in host cells through its spike protein (S protein) [110]. Mutations in S protein affect viral phagocytosis and have a significant impact on infectivity and mortality [111]. A variety of COVID-19 vaccines received emergency use authorizations; they can be classified into four main types: (1) mRNA vaccines, represented by BNT162b2 (Pfizer-BioNTech) and mRNA-1273 (Moderna); (2) adenovirus vector-based vaccines, represented by Ad26COVS1 (Johnson and Johnson); (3) protein subunit vaccines, represented by NVX-CoV2373 (Novavax); and (4) whole virus vaccines, including inactivated vaccines—such as CoronaVac from Sinovac Biotech—and live attenuated vaccines—such as COVI-VAC from Codagenix [112,113]. As of 26 February 2024, a total of 13.59 billion doses of the COVID-19 vaccines have been administered [114]; therefore, they constitute a powerful tool for effectively reducing mortality and severe illness. However, there have been multiple reports of adverse ocular reactions following administration of COVID-19 vaccines and booster doses [113,115,116]. Ocular side effects include neuropathy [111,117], uveitis [118], primary inflammatory choriocapillaropathy (PICCP) [119,120], frosted branch angiitis [121], and other immune-related eye diseases. Ocular side effects have mainly been observed after mRNA vaccine (BNT162b2 and mRNA-1273) application.

One hypothesis for eye disease associated with mRNA vaccines is due to allergic reactions triggered by PEG. mRNA vaccines primarily consist of mRNA fragments coding for a single SARS-CoV-2 antigen (such as the S protein antigen) enclosed in a shell and delivered through lipid nanoparticles. The mRNA in the BNT162b2 and mRNA-1273 vaccines was encapsulated in two novel types of polyethylene glycol (PEG) nanoparticles. Noteworthily, several studies have reported the potential of PEG to induce allergic reactions, indicating that patients with a history of PEG-induced allergic reactions [48] and PEG-induced complement activation [122] have IgE antibodies in their bodies, which may subsequently trigger ocular inflammation.

Although the precise mechanisms underlying PEG-induced hypersensitivity reactions have not been fully elucidated, an increasing body of evidence suggests that complement activation plays a crucial role in the development of such hypersensitivity reactions [123]. The complement system plays a significant role in the innate immune defense against foreign antigens [124], and its activation is tightly regulated by the assembly of surface proteins to prevent harm to normal tissues. However, excessive complement activation, observed in certain autoimmune diseases, can lead to severe damage to multiple organs [125]. In recent years, numerous studies have indicated that in healthy individuals frequently exposed to PEG-containing cleansers or cosmetics, PEG molecules can penetrate inflammatory sites and come into contact with inflammatory cells, thereby triggering the formation of anti-PEG antibodies [126,127,128,129]. The presence of PEG antibodies is closely linked to complement reactions. In the case of PEGylated liposomes and PEG-G-CSF, anti-PEG antibodies play a crucial role in PEG-induced complement activation-related pseudoallergy (CARPA) through the classical complement pathway [130,131]. Additionally, the dual-hit theory provides another mechanism to explain PEG-induced hypersensitivity reactions, suggesting that PEGylated nanocarrier surfaces interact with immune-regulatory cells, such as macrophages and mast cells, through specific surface receptors, stimulating signal transduction networks that mediate secretion responses [122,132] (Table 1).

### 3.6. Type 1 IFN Activation and the Inflammatory Response of Retinal Pigment Epithelial (RPE) Cells

An alternative hypothesis for ocular disease associated with mRNA vaccines is that RNA triggers Type 1 IFN activation, which subsequently leads to a validation response in retinal pigment epithelial (RPE) cells. The role of Type 1 IFN in host defense is well established. An increasing body of evidence suggests that Type 1 IFN plays a crucial role not only in host defense against infections but also in sterile inflammation, particularly in autoimmune diseases, such as systemic lupus erythematosus, multiple sclerosis Type 1 IFN, Sjogren’s syndrome [133], atherosclerosis [134], and Alzheimer’s disease [135]. Interestingly, studies have shown that Type 1 IFN also plays an important role in age-related diseases such as age-related macular degeneration (AMD) [136]

Following mRNA vaccine administration, inflammatory cells, including neutrophils, dendritic cells (DCs), and macrophages, are recruited to the injection site [112]. These inflammatory cells activate the secretion of Type 1 IFN, which in turn activates hundreds of IFN-stimulated genes, thereby blocking viral transcription and degrading viral RNA. Simultaneously, antigen-presenting cells are activated, transmitting viral signals to T cells, thereby activating inflammation and cytotoxic mediators associated with CD4+ and CD8+ T-cell differentiation. CD4+ helper T cells can promote B-cell differentiation into antibody-secreting plasma cells, leading to the production of specific antibodies against the novel coronavirus [137].

**Figure 4:**
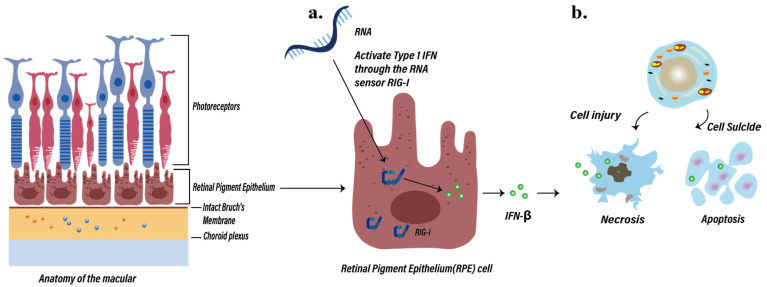
Possible inflammatory response pathway in retinal pigment epithelial (RPE) cells induced by COVID-19 mRNA vaccines. (**a**) RPE cells respond to intracellular RNA through the RNA sensor RIG-I (DDX58), enhancing Type 1 IFN response and increasing IFN-β levels. (**b**) Type 1 IFN activation may also initiate cell death and apoptosis pathways by upregulating a spectrum of interferon-stimulated genes. IFN, interferon; RIG-I, Retinoic Acid-Inducible Gene I.

However, the activation of Type 1 IFN may also be implicated in the inflammatory response of RPE cells, leading to the occurrence of vaccine-related ocular side effects (Figure 4). RIG-I (DExD/H-box helicase 58, DDX58) is a dsRNA helicase that plays a crucial role in RNA sensing [138]. Numerous studies have confirmed that RIG-I is widely expressed in most cells, including the RPE cells [139], and RNA can directly activate the Type 1 IFN response in RPE cells through RIG-I [140,141]. In addition, studies have shown that in RPE cells of patients with AMD accompanied by geographic atrophy (GA), Type 1 IFN is activated and enriched [139]. In 2021, a study by Schustak et al. [136] on the nucleic acid-sensing mechanism in RPE cells validated this hypothesis [127]. They utilized the A549 RIG-I KO cell line to explore the expression of RIG-I in the RPE cells of patients with AMD. The results revealed a significant increase in RIG-I mRNA expression in patients with AMD and GA compared to the control group, particularly in the retinal ganglion cells (RGCs), outer nuclear layer (ONL), inner nuclear layer (INL), and RPE cell layers. Therefore, it can be inferred that RPE cells can respond to intracellular RNA through the RNA sensor RIG-I (DDX58), enhancing the Type 1 IFN response. This is primarily manifested by an increase in IFN-β levels in patient RPE cells. Type 1 IFN activation may initiate cell death pathways by upregulating a spectrum of IFN-stimulated genes, including mixed lineage kinase domain-like pseudokinase (MLKL), CASP7/8 (involved in apoptosis), tumor necrosis factor (TNF) superfamily member 10 (TNFSF10, also known as TRAIL), and gasdermin-D (GSDMD; involved in pyroptosis) [136,142,143,144,145,146]. This activation can further induce cell degeneration/death in the retina, rendering cells sensitive to secondary damage, and ultimately leading to the loss of RPE cell barrier function and triggering inflammatory changes in the eye [119] (Figure 4 and Table 1).

### 3.7. Free Extracellular RNA

Extracellular free RNA is also one of the hypothesized contributors to ocular inflammation after RNA vaccination. Fowler et al. reported the case of a 33-year-old male who experienced blurred vision and color vision loss in the right eye 69 h after receiving the first dose of the BNT162b2 mRNA COVID-19 vaccine [50]. OCT angiography revealed a decreased choroidal flow signal, leading to a diagnosis of central serous retinopathy (CSR) following comprehensive assessment. Given the rapid onset of ocular symptoms post-immunization and the absence of typical CSR risk factors, such as a history of exogenous steroid use, psychological stress, or Type A personality, a potential association between patient presentation and vaccine administration may be suspected.

It has been demonstrated that naked extracellular RNA can increase endothelial cell permeability, leading to increased choroidal vascular permeability [147]. Additionally, extracellular RNA, exposed to blood components, can promote blood coagulation and thrombus formation [148]. The pathophysiology of CSR is closely associated with choroidal hyperpermeability and thickening. Studies have indicated that choroidal staining in the retinal areas correlates with delayed choroidal perfusion, suggesting choroidal lobular ischemia with associated venous dilation [149], which is consistent with the OCT angiography findings reported by Fowler et al. and other case studies [149]. Furthermore, elevated plasminogen activator inhibitor-1 levels in patients with CSR provide a basis for the thrombotic mechanism underlying these vascular changes [150] (Table 1).

### 3.8. Specific Components

#### Thimerosal

Influenza vaccine-induced eye inflammation has been widely reported, with most studies attributing it to the addition of adjuvants and direct infection from inactivated vaccines. However, the H1N1 vaccine (Vaxigrip, by Sanofi Pasteur) does not contain any adjuvants. It contains only trace amounts of thimerosal, formaldehyde, and Triton X-100, and minute amounts of neomycin [51]. Mutter et al. found that, in the United States, routine vaccination of 6-month-old infants with vaccines containing thimerosal (49.6% ethyl mercury) was associated with a tenfold increase in the incidence of Kawasaki disease [151]. Since 1990, the Centers for Disease Control and Prevention (CDC) has received reports from 88 patients diagnosed with Kawasaki disease shortly after vaccination. The mechanisms underlying this phenomenon may be related to the effects of mercury on the immune system. Mercury can tightly bind to thiols in tissues and alter their molecular structures. T lymphocytes may erroneously identify metal-modified cells as foreign, triggering an autoimmune response that attacks the body tissues [152] (Table 1).

## 4. Conclusions

Vaccines play an indispensable role in preventing infectious diseases and are distinct from the antimicrobial drugs used to treat infections, as vaccines primarily target healthy individuals. Consequently, adverse reactions following vaccination are closely correlated, with particular attention being focused on vaccine-related ocular side effects. Through in-depth research on the immunological and molecular mechanisms of vaccines, we can gain a better understanding of the occurrence and development of these side effects.

First, the ocular side effects triggered by vaccines may be linked to the activation of the immune system. Attenuated yet active vaccines typically contain antigens capable of directly infecting the body, leading to the corresponding side effects. Some attenuated live vaccines and adjuvants in inactivated vaccines may induce autoimmune diseases known as Shoenfeld’s syndrome. Type III and IV hypersensitivity reactions may play a role in postvaccination ocular inflammation, where excessive or aberrant immune system activation may result in ocular tissue damage, leading to a variety of ocular side effects. Additionally, molecular mimicry triggered by certain vaccine components that share homology with human tissues may explain some instances of ocular inflammation.

Second, with the global impact of SARS-CoV-2 on human health, numerous reports on COVID-19 vaccine-related ocular side effects have been published. The underlying mechanisms might be different from the aforementioned and be related to the constituent PEG in mRNA vaccines; alternatively, it could be due to RNA directly activating the RIG-I sensor, inducing a Type 1 IFN response in RPE cells, thereby upregulating a cascade of interferon-stimulated reaction, ultimately initiating the death pathway in these RPE cells.

It is important to note that the exact mechanisms of ocular-related diseases induced by vaccines have not been fully elucidated. The mechanisms reported in this study, including AS01B-induced ocular inflammation, PEG-induced allergic reactions, Type 1 IFN activation, and the inflammatory response of retinal pigment epithelial (RPE) cells, free extracellular RNA, and specific compounds like thimerosal, are still in the hypothesis stage and require further basic research for confirmation.

Building on a deeper understanding of the potential immunological and molecular mechanisms of vaccines is crucial to enhance monitoring and assessment to ensure vaccine safety and efficacy. We suggest implementing personalized medical monitoring and intervention, especially for those who are susceptible to ocular side effects, including but not limited to individuals with specific genetic backgrounds, known immune system abnormalities or medical histories, those already experiencing eye health issues, as well as individuals who may exhibit different responses to vaccines due to factors such as elderly people, women, or those with other health considerations. Furthermore, individuals who have experienced ocular side effects from vaccination in the past should also undergo personalized monitoring and intervention. Through further research and scientific collaboration, we can better comprehend the impact of vaccines on ocular health and provide comprehensive guidance for the future development and application of vaccines. However, it is noteworthy that vaccine side effects do not outweigh their advantages in preventing economically, socially, and domestically burdensome diseases. Overall, maintaining a balanced perspective on vaccines, while considering their potential side effects, is imperative.

## Figures and Tables

**Table 1:** Vaccine-associated ocular inflammation.

Underlying Mechanism		Ocular Inflammation	Licensed Vaccine *	Reference
Direct infection		ARN, MEWDS, keratitis, iris heterochromia, uveitis	MMR vaccine (Priorix, strain Wistar RA 27/3; GlaxoSmithKline, Brentford, Middlesex), nasal spray influenza vaccine ^, rotavirus vaccines (RotaTeq^®^ by Merck & Co., Inc., Kenilworth, NJ, USA), yellow fever vaccine ^, live attenuated varicella vaccine (Zostavax^®^ by Merck & Co., Inc., Kenilworth, NJ, USA)	[15,16,17,18,20,21,22,31]
ASIA	Aluminum-related	NMO	HPV vaccine (Gardasil, Merck & Co., Inc., USA)	[32,33]
	Interactions between adjuvants and adaptive immune components	Uveitis, VKH	HBV vaccine (Recombivax HB and Engerix-B)	[24,30]
	AS01B induced	Uveitis, ARN, HZO reactivation, stromal keratitis	VZV vaccine (Shingrix by GlaxoSmithKline, Brentford, UK)	[34,35,36,37,38]
Hypersensitivity		Optic neuritis, NMOSD, MEWDS, ERD	Rabies vaccine (Rabipur^®^ by Hoechst Marion Roussel Ltd, Ankleshwar, Gujarat), influenza vaccines (split, inactivated virions of swine influenza A New Jersey and influenza B Victoria lineages), MMR vaccine ^, HPV vaccine (Gardasil, Merck & Co., Inc., Kenilworth, NJ, USA)	[39,40,41,42,43,44]
Molecular mimicry		Optic neuritis, uveitis, APMPPE, TINU syndrome	HPV vaccine (Gardasil, Merck & Co., Inc., Kenilworth, NJ, USA), purified hepatitis B vaccine ^	[41,42,45,46,47]
PEG-induced allergic reactions		Optic neuropathy, VKH, MEWDS, AMN	COVID-19 vaccines (BNT163b2 by Pfizer-BioNTech, BioNTech Manufacturing GmbH, Berlin, Germany, and mRNA-1273, by Moderna Biotech, Madrid, Spain)	[48]
Activation of Type 1 IFN and inflammatory response of RPE cells		MEWDS		[49]
Free extracellular mRNA		CSR		[50]
Specific components (thimerosal)		Optic neuritis	Influenza (Vaxigrip by Sanofi Pasteur, Lyon, France)	[51]

* Trade Names as specified by case report or case series. ^ Trade names for vaccines unspecified or unknown in case report or case series. Abbreviations: AMN, acute macular neuroretinopathy; APMPPE, acute posterior multifocal placoid pigment epitheliopathy; ARN, acute retinal necrosis; CSR, central serous retinopathy; ERD, exudative retinal detachment; HBV, hepatitis B virus; HPV, human papillomavirus; HZO, herpes zoster ophthalmicus; MEWDS = multiple evanescent white dot syndrome; MMR, measles–mumps–rubella; NMO, neuromyelitis optica; NMOSD, neuromyelitis optica spectrum disorder; RPE cells, retinal pigment epithelial cells; TINU, tubulointerstitial nephritis and uveitis; VKH, Vogt–Koyanagi–Harada syndrome; VZV = varicella zoster virus; YF, yellow fever; ASIA, autoimmune/inflammatory syndrome induced by adjuvants; PEG, polyethylene glycol; CSR, central serous retinopathy; RPE, retinal pigment epithelial; IFN, Interferon.

## Data Availability

All data related to this study are presented and published here.

## References

[1] Watad A., Bragazzi N.L., McGonagle D., Adawi M., Bridgewood C., Damiani G., Alijotas-Reig J., Esteve-Valverde E., Quaresma M., Amital H. (2019). Autoimmune/inflammatory syndrome induced by adjuvants (ASIA) demonstrates distinct autoimmune and autoinflammatory disease associations according to the adjuvant subtype: Insights from an analysis of 500 cases. Clin. Immunol..

[2] Tervaert J.W.C., Martinez-Lavin M., Jara L.J., Halpert G., Watad A., Amital H., Shoenfeld Y. (2023). Autoimmune/inflammatory syndrome induced by adjuvants (ASIA) in 2023. Autoimmun. Rev..

[3] Vera-Lastra O., Medina G., Cruz-Dominguez M.d.P., Ramirez P., Gayosso-Rivera J.A., Anduaga-Dominguez H., Lievana-Torres C., Jara L.J. (2012). Human adjuvant disease induced by foreign substances: A new model of ASIA (Shoenfeld’s syndrome). Lupus.

[4] Bragazzi N.L., Watad A., Amital H., Shoenfeld Y. (2017). Debate on vaccines and autoimmunity: Do not attack the author, yet discuss it methodologically. Vaccine.

[5] Elisha E., Guetzkow J., Shir-Raz Y., Ronel N. (2024). Suppressing Scientific Discourse on Vaccines? Self-perceptions of researchers and practitioners. HEC Forum.

[6] Salmon D.A., Proschan M., Forshee R., Gargiullo P., Bleser W., Burwen D.R., Cunningham F., Garman P., Greene S.K., Lee G.M. (2013). Association between Guillain-Barré syndrome and influenza A (H1N1) 2009 monovalent inactivated vaccines in the USA: A meta-analysis. Lancet.

[7] Miravalle A., Biller J., Schnitzler E., Bonwit A. (2010). Neurological complications following vaccinations. Neurol. Res..

[8] Cecinati V., Principi N., Brescia L., Giordano P., Esposito S. (2013). Vaccine administration and the development of immune thrombocytopenic purpura in children. Hum. Vaccines Immunother..

[9] Benage M., Fraunfelder F.W. (2016). Vaccine-Associated Uveitis. Mo. Med..

[10] Agarwal M., Dutta Majumder P., Babu K., Konana V.K., Goyal M., Touhami S., Stanescu-Segall D., Bodaghi B. (2020). Drug-induced uveitis: A review. Indian J. Ophthalmol..

[11] Cheng J.Y., Margo C.E. (2022). Ocular adverse events following vaccination: Overview and update. Surv. Ophthalmol..

[12] Zou Y., Kamoi K., Zong Y., Zhang J., Yang M., Ohno-Matsui K. (2023). Ocular Inflammation Post-Vaccination. Vaccines.

[13] Minor P.D. (2015). Live attenuated vaccines: Historical successes and current challenges. Virology.

[14] Oxman M.N., Levin M.J., Johnson G., Schmader K., Straus S., Gelb L., Arbeit R., Simberkoff M., Gershon A., Davis L. (2005). A vaccine to prevent herpes zoster and postherpetic neuralgia in older adults. N. Engl. J. Med..

[15] Grillo A., Fraunfelder F.W. (2017). Keratitis in association with herpes zoster and varicella vaccines. Drugs Today.

[16] Heath G., Depledge D.P., Brown J.R., Hale A.D., Tutil H., Williams R., Breuer J. (2017). Acute Retinal Necrosis Caused by the Zoster Vaccine Virus. Clin. Infect. Dis. Off. Publ. Infect. Dis. Soc. Am..

[17] Gonzales J.A., Levison A.L., Stewart J.M., Acharya N.R., Margolis T.P. (2012). Retinal necrosis following varicella-zoster vaccination. Arch. Ophthalmol..

[18] Ferrini W., Aubert V., Balmer A., Munier F.L., Abouzeid H. (2013). Anterior uveitis and cataract after rubella vaccination: A case report of a 12-month-old girl. Pediatrics.

[19] Jampol L.M., Sieving P.A., Pugh D., Fishman G.A., Gilbert H. (1984). Multiple evanescent white dot syndrome. I. Clinical findings. Arch. Ophthalmol..

[20] Stangos A., Zaninetti M., Petropoulos I., Baglivo E., Pournaras C. (2006). Multiple evanescent white dot syndrome following simultaneous hepatitis-A and yellow fever vaccination. Ocul. Immunol. Inflamm..

[21] Fine L., Fine A., Cunningham E.T. (2001). Multiple evanescent white dot syndrome following hepatitis a vaccination. Arch. Ophthalmol..

[22] Goyal S., Nazarian S.M., Thayi D.R., Hammond F., Petrovic V. (2013). Multiple evanescent white dot syndrome following recent influenza vaccination. Can. J. Ophthalmol..

[23] Watad A., Quaresma M., Brown S., Cohen Tervaert J.W., Rodríguez-Pint I., Cervera R., Perricone C., Shoenfeld Y. (2017). Autoimmune/inflammatory syndrome induced by adjuvants (Shoenfeld’s syndrome)—An update. Lupus.

[24] Sood A.B., O’Keefe G., Bui D., Jain N. (2019). Vogt-Koyanagi-Harada Disease Associated with Hepatitis B Vaccination. Ocul. Immunol. Inflamm..

[25] Watad A., Quaresma M., Bragazzi N.L., Cervera R., Tervaert J.W.C., Amital H., Shoenfeld Y. (2018). The autoimmune/inflammatory syndrome induced by adjuvants (ASIA)/Shoenfeld’s syndrome: Descriptive analysis of 300 patients from the international ASIA syndrome registry. Clin. Rheumatol..

[26] Awate S., Babiuk L.A., Mutwiri G. (2013). Mechanisms of action of adjuvants. Front. Immunol..

[27] Marciani D.J. (2003). Vaccine adjuvants: Role and mechanisms of action in vaccine immunogenicity. Drug Discov. Today.

[28] Koenig H.C., Sutherland A., Izurieta H.S., McGonagle D. (2011). Application of the immunological disease continuum to study autoimmune and other inflammatory events after vaccination. Vaccine.

[29] McGonagle D., McDermott M.F. (2006). A proposed classification of the immunological diseases. PLoS Med..

[30] Fraunfelder F.W., Suhler E.B., Fraunfelder F.T. (2010). Hepatitis B vaccine and uveitis: An emerging hypothesis suggested by review of 32 case reports. Cutan. Ocul. Toxicol..

[31] Islam S.M., El-Sheikh H.F., Tabbara K.F. (2000). Anterior uveitis following combined vaccination for measles, mumps and rubella (MMR): A report of two cases. Acta Ophthalmol. Scand..

[32] Menge T., Cree B., Saleh A., Waterboer T., Berthele A., Kalluri S.R., Hemmer B., Aktas O., Hartung H.-P., Methner A. (2012). Neuromyelitis optica following human papillomavirus vaccination. Neurology.

[33] Chang H., Lee H.L., Yeo M., Kim J.S., Shin D.I., Lee S.S., Lee S.H. (2016). Recurrent optic neuritis and neuromyelitis optica-IgG following first and second human papillomavirus vaccinations. Clin. Neurol. Neurosurg..

[34] Heydari-Kamjani M., Vante I., Uppal P., Demory Beckler M., Kesselman M.M. (2019). Uveitis Sarcoidosis Presumably Initiated after Administration of Shing rix Vaccine. Cureus.

[35] Jabbour S., Shekhawat N.S., Chen A., Woreta F.A. (2021). Presumed Herpes Zoster Ophthalmicus Reactivation Following Recombinant Zoster Vaccination. Cornea.

[36] Richards P.J., Wingelaar M.J., Armbrust K.R., Kopplin L.J. (2021). Uveitis reactivation following recombinant zoster vaccination. Am. J. Ophthalmol. Case Rep..

[37] Chen R.I., Deaner J.D., Srivastava S.K., Lowder C.Y. (2020). Acute retinal necrosis following recombinant subunit varicella-zoster virus vaccine. Am. J. Ophthalmol. Case Rep..

[38] Lehmann A., Matoba A. (2018). Reactivation of Herpes Zoster Stromal Keratitis After HZ/su Adjuvanted Herpes Zoster Subunit Vaccine. Ophthalmology.

[39] Saxena R., Sethi H.S., Rai H.K., Menon V. (2005). Bilateral neuro-retinitis following chick embryo cell anti-rabies vaccination—A case report. BMC Ophthalmol..

[40] Stevenson V.L., Acheson J.F., Ball J., Plant G.T. (1996). Optic neuritis following measles/rubella vaccination in two 13-year-old children. Br. J. Ophthalmol..

[41] Fried M., Conen D., Conzelmann M., Steinemann E. (1987). Uveitis after hepatitis B vaccination. Lancet.

[42] Sawai T., Shimizu M., Sakai T., Yachie A. (2016). Tubulointerstitial Nephritis and Uveitis Syndrome Associated with Human Papillomavirus Vaccine. J. Pediatr. Ophthalmol. Strabismus.

[43] Gupta V., Bandyopadhyay S., Bapuraj J.R., Gupta A. (2004). Bilateral Optic Neuritis Complicating Rabies Vaccination. Retina.

[44] Yang J.S., Chen C.L., Hu Y.Z., Zeng R. (2018). Multiple evanescent white dot syndrome following rabies vaccination: A case report. BMC Ophthalmol..

[45] Chen Y.-H., Chang Y.-H., Lee Y.-C. (2014). Panuveitis following administration of quadrivalent human papillomavirus vaccine. Tzu Chi Med. J..

[46] Khalifa Y.M., Monahan P.M., Acharya N.R. (2010). Ampiginous choroiditis following quadrivalent human papilloma virus vaccine. Br. J. Ophthalmol..

[47] Kraemer L.S., Montgomery J.R., Baker K.M., Colyer M.H. (2022). Acute posterior multifocal placoid pigment epitheliopathy after immunization with multiple vaccines. Retin. Cases Brief Rep..

[48] Zhou Z.-H., Stone C.A., Jakubovic B., Phillips E.J., Sussman G., Park J., Hoang U., Kirshner S.L., Levin R., Kozlowski S. (2021). Anti-PEG IgE in anaphylaxis associated with polyethylene glycol. J. Allergy Clin. Immunol. Pract..

[49] Yasuda E., Matsumiya W., Maeda Y., Kusuhara S., Nguyen Q.D., Nakamura M., Hara R. (2022). Multiple evanescent white dot syndrome following BNT162b2 mRNA COVID-19 vaccination. Am. J. Ophthalmol. Case Rep..

[50] Fowler N., Mendez Martinez N.R., Pallares B.V., Maldonado R.S. (2021). Acute-onset central serous retinopathy after immunization with COVID-19 mRNA vaccine. Am. J. Ophthalmol. Case Rep..

[51] Rubinov A., Beiran I., Krasnitz I., Miller B. (2012). Bilateral optic neuritis after inactivated influenza vaccination. Isr. Med. Assoc. J. IMAJ.

[52] Gherardi R.K., Crépeaux G., Authier F.-J. (2019). Myalgia and chronic fatigue syndrome following immunization: Macrophag ic myofasciitis and animal studies support linkage to aluminum adjuvan t persistency and diffusion in the immune system. Autoimmun. Rev..

[53] Verdier F., Burnett R., Michelet-Habchi C., Moretto P., Fievet-Groyne F., Sauzeat E. (2005). Aluminium assay and evaluation of the local reaction at several time points after intramuscular administration of aluminium containing vaccines in the Cynomolgus monkey. Vaccine.

[54] Authier F.-J., Sauvat S., Christov C., Chariot P., Raisbeck G., Poron M.-F., Yiou F., Gherardi R. (2006). Al(OH)_3_-adjuvanted vaccine-induced macrophagic myofasciitis in rats is influenced by the genetic background. Neuromuscul. Disord..

[55] Priest N., Newton D., Day J., Talbot R., Warner A. (1995). Human metabolism of aluminium-26 and gallium-67 injected as citrates. Hum. Exp. Toxicol..

[56] Flarend R.E., Hem S.L., White J.L., Elmore D., Suckow M.A., Rudy A.C., Dandashli E.A. (1997). In vivo absorption of aluminium-containing vaccine adjuvants using 26Al. Vaccine.

[57] Banstola A., Reynolds J.N.J. (2022). The Sheep as a Large Animal Model for the Investigation and Treatment of Human Disorders. Biology.

[58] González J.M., Figueras L., Ortega M.E., Lozano M., de Arcaute M.R., Royo R., Cebrián L.M., Ferrer L.M., Fariñas F., de Jalón J.A. (2010). Possible adverse reactions in sheep after vaccination with inactivated BTV vaccines. Vet. Rec..

[59] Gunasekaran M., Chatterjee P.K., Shih A., Imperato G.H., Addorisio M., Kumar G., Lee A., Graf J.F., Meyer D., Marino M. (2018). Immunization elicits antigen-specific antibody sequestration in dorsal root ganglia sensory neurons. Front. Immunol..

[60] Gilbert M.R., Harding B.L., Hoffman P.N., Griffin J.W., Price D.L., Troncoso J.C. (1992). Aluminum-induced neurofilamentous changes in cultured rat dorsal root ganglia explants. J. Neurosci..

[61] Khalifa Y.M., Jacoby R.M., Margolis T.P. (2010). Exacerbation of zoster interstitial keratitis after zoster vaccination in an adult. Arch. Ophthalmol..

[62] Jastrzebski A., Brownstein S., Ziai S., Saleh S., Lam K., Jackson W.B. (2017). Reactivation of herpes zoster keratitis with corneal perforation after zoster vaccination. Cornea.

[63] Lal H., Cunningham A.L., Godeaux O., Chlibek R., Diez-Domingo J., Hwang S.J., Levin M.J., McElhaney J.E., Poder A., Puig-Barberà J. (2015). Efficacy of an adjuvanted herpes zoster subunit vaccine in older adults. N. Engl. J. Med..

[64] Bharucha T., Ming D., Breuer J. (2017). A critical appraisal of ‘Shingrix’, a novel herpes zoster subunit vaccine (HZ/Su or GSK1437173A) for varicella zoster virus. Hum. Vaccines Immunother..

[65] Tilton R.G., Chang K., Corbett J.A., Misko T.P., Currie M.G., Bora N.S., Kaplan H.J., Williamson J.R. (1994). Endotoxin-induced uveitis in the rat is attenuated by inhibition of nitric oxide production. Investig. Ophthalmol. Vis. Sci..

[66] Newman M.J., Wu J.Y., Gardner B.H., Anderson C.A., Kensil C.R., Recchia J., Coughlin R.T., Powell M.F. (1997). Induction of cross-reactive cytotoxic T-lymphocyte responses specific for HIV-1 gp120 using saponin adjuvant (QS-21) supplemented subunit vaccine formulations. Vaccine.

[67] Guy B. (2007). The perfect mix: Recent progress in adjuvant research. Nat. Rev. Microbiol..

[68] Chlibek R., Bayas J.M., Collins H., de la Pinta M.L., Ledent E., Mols J.F., Heineman T.C. (2013). Safety and immunogenicity of an AS01-adjuvanted varicella-zoster virus subunit candidate vaccine against herpes zoster in adults ≥ 50 years of age. J. Infect. Dis..

[69] Chlibek R., Smetana J., Pauksens K., Rombo L., Van den Hoek J.A., Richardus J.H., Plassmann G., Schwarz T.F., Ledent E., Heineman T.C. (2014). Safety and immunogenicity of three different formulations of an adjuvanted varicella-zoster virus subunit candidate vaccine in older adults: A phase II, randomized, controlled study. Vaccine.

[70] Landrigan P.J. (1973). Neurologic Disorders Following Live Measles-Virus Vaccination. JAMA.

[71] Dyck P.J., Daube J., O’Brien P., Pineda A., Low P.A., Windebank A.J., Swanson C. (1986). Plasma exchange in chronic inflammatory demyelinating polyradiculoneur opathy. N. Engl. J. Med..

[72] Behan P.O., Moore M.J., Lamarche J.B. (1973). Acute Necrotizing Hemorrhagic Encephalopathy. Postgrad. Med..

[73] Poser C.M., Alter M., Currier R.D., Hunter S.E. (1979). Common Demyelinating and Degenerative Diseases and Extrapyramidal Disorders—Panel 4. Arch. Neurol..

[74] Reik L. (1980). Disseminated vasculomyelinopathy: An immune complex disease. Ann. Neurol..

[75] Drachman D.A., Paterson P.Y., Berlin B.S., Roguska J. (1970). Immunosuppression and the Guillain-Barré Syndrome. Arch. Neurol..

[76] Goust J.M., Chenais F.O., Carnes J.E., Hames C.G., Fudenberg H.H., Hogan E.L. (1978). Abnormal T cell subpopulations and circulating immune complexes in the Guillain-Barré syndrome and multiple sclerosis. Neurology.

[77] Hodson A.K., Doughty R.A., Norman M.E. (1978). Acute Encephalopathy, Streptococcal Infection, and Cryoglobulinemia. Arch. Neurol..

[78] Sergent J.S., Lockshin M.D., Christian C.L., Gocke D.J. (1976). Vasculitis with hepatitis B antigenemia: Long-term observation in nine patients. Medicine.

[79] Chakravarty A. (2001). Neurologic illness following post-exposure prophylaxis with purifiled chick embryo cell antirabies vaccine. J. Assoc. Physicians India.

[80] Waisbren B.A. (2008). Acquired autoimmunity after viral vaccination is caused by molecular mimicry and antigen complimentarity in the presence of an immunologic adjuvant and specific HLA patterns. Med. Hypotheses.

[81] Fujinami R.S., Oldstone M.B.A. (1985). Amino Acid Homology Between the Encephalitogenic Site of Myelin Basic Protein and Virus: Mechanism for Autoimmunity. Science.

[82] Wucherpfennig K.W., Strominger J.L. (1995). Molecular mimicry in T cell-mediated autoimmunity: Viral peptides activate human T cell clones specific for myelin basic protein. Cell.

[83] Westall F.C., Root-Bernstein R.S. (1983). An explanation of prevention and suppression of experimental allergic encephalomyelitis. Mol. Immunol..

[84] Nakamura S., Yamakawa T., Sugita M., Kijima M., Ishioka M., Tanaka S., Ohno S. (1994). The role of tumor necrosis factor-alpha in the induction of experimental autoimmune uveoretinitis in mice. Investig. Ophthalmol. Vis. Sci..

[85] Yokochi T., Fujii Y., Nakashima I., Asai J., Kiuchi M., Kojima K., Kato N. (1993). A murine model of experimental autoimmune lens-induced uveitis using Klebsiella O3 lipopolysaccharide as a potent immunological adjuvant. Int. J. Exp. Pathol..

[86] de Vos A.F., Klaren V.N., Kijlstra A. (1994). Expression of multiple cytokines and IL-1RA in the uvea and retina during endotoxin-induced uveitis in the rat. Investig. Ophthalmol. Vis. Sci..

[87] Charteris D.G., Lightman S.L. (1993). In vivo lymphokine production in experimental autoimmune uveoretinitis. Immunology.

[88] Merino G., Fujino Y., Hanashiro R.K. (1998). Lipoteichoic acid as an inducer of acute uveitis in the rat. Investig. Ophthalmol. Vis. Sci..

[89] Natale C., Giannini T., Lucchese A., Kanduc D. (2000). Computer-assisted analysis of molecular mimicry between human papillomavirus 16 E7 oncoprotein and human protein sequences. Immunol. Cell Biol..

[90] Garip A., Diedrichs-Möhring M., Thurau S.R., Deeg C.A., Wildner G. (2009). Uveitis in a patient treated with Bacille-Calmette-Guérin: Possible antigenic mimicry of mycobacterial and retinal antigens. Ophthalmology.

[91] Gass J.D.M. (1968). Acute posterior multifocal placoid pigment epitheliopathy. Arch. Ophthalmol..

[92] Reichhart M.D., Patterson M.C., Borruat F.-X. (2018). Chapter 37—Acute Posterior Multifocal Placoid Pigment Epitheliopathy. Uncommon Causes of Stroke.

[93] Escott S., Tarabishy A.B., Davidorf F.H. (2013). Multifocal choroiditis following simultaneous hepatitis A, typhoid, and yellow fever vaccination. Clin. Ophthalmol..

[94] Wolf M.D., Folk J.C., Panknen C.A., Goeken N.E. (1990). HLA-B7 and HLA-DR2 antigens and acute posterior multifocal placoid pigment epitheliopathy. Arch. Ophthalmol..

[95] Yang X., Garner L.I., Zvyagin I.V., Paley M.A., Komech E.A., Jude K.M., Zhao X., Fernandes R.A., Hassman L.M., Paley G.L. (2022). Autoimmunity-associated T cell receptors recognize HLA-B*27-bound pept ides. Nature.

[96] Baxter K.R., Opremcak E.M. (2013). Panretinal acute multifocal placoid pigment epitheliopathy: A novel posterior uveitis syndrome with HLA-A3 and HLA-C7 association. J. Ophthalmic Inflamm. Infect..

[97] Stepanov A., Feuermannová A., Studnička J., Hejsek L., Burova M., Jirásková N., Rozsíval P. (2014). Acute posterior multifocal placoid pigment epitheliopathy-case report. Ceska A Slov. Oftalmol. Cas. Ceske Oftalmol. Spol. A Slov. Oftalmol. Spol..

[98] Amaro D., Carreño E., Steeples L.R., Oliveira-Ramos F., Marques-Neves C., Leal I. (2020). Tubulointerstitial nephritis and uveitis (TINU) syndrome: A review. Br. J. Ophthalmol..

[99] Mackensen F., David F., Schwenger V., Smith L.K., Rajalingam R., Levinson R.D., Austin C.R., Houghton D., Martin T.M., Rosenbaum J.T. (2011). HLA-DRB1* 0102 is associated with TINU syndrome and bilateral, sudden-onset anterior uveitis but not with interstitial nephritis alone. Br. J. Ophthalmol..

[100] Reddy A.K., Hwang Y.-S., Mandelcorn E.D., Davis J.L. (2013). HLA-DR, DQ class II DNA typing in pediatric panuveitis and tubulointer stitial nephritis and uveitis. Am. J. Ophthalmol..

[101] Clive D.M., Vanguri V.K. (2017). The Syndrome of Tubulointerstitial Nephritis with Uveitis (TINU). Am. J. Kidney Dis..

[102] Tanaka H., Suzuki K., Nakahata T., Tateyama T., Waga S., Ito E. (2001). Repeat renal biopsy in a girl with tubulointerstitial nephritis and uveitis syndrome. Pediatr. Nephrol..

[103] Dummer C.D., Carpio V.N., Gonçalves L.F.S., Manfro R.C., Veronese F.V. (2012). FOXP3+ regulatory T cells: From suppression of rejection to induction of renal allograft tolerance. Transpl. Immunol..

[104] Rytkönen S.H., Kulmala P., Autio-Harmainen H., Arikoski P., Endén K., Kataja J., Karttunen T., Nuutinen M., Jahnukainen T. (2018). FOXP3+ T cells are present in kidney biopsy samples in children with tubulointerstitial nephritis and uveitis syndrome. Pediatr. Nephrol..

[105] Shimazaki K., Jirawuthiworavong G.V., Nguyen E.V., Awazu M., Levinson R.D., Gordon L.K. (2008). Tubulointerstitial Nephritis and Uveitis Syndrome: A Case with an Autoimmune Reactivity Against Retinal and Renal Antigens. Ocul. Immunol. Inflamm..

[106] Tan Y., Yu F., Qu Z., Su T., Xing G.-Q., Wu L.-H., Wang F.-M., Liu G., Yang L., Zhao M.-H. (2011). Modified C-reactive protein might be a target autoantigen of TINU synd rome. Clin. J. Am. Soc. Nephrol..

[107] Li C., Su T., Chu R., Li X., Yang L. (2014). Tubulointerstitial nephritis with uveitis in Chinese adults. Clin. J. Am. Soc. Nephrol..

[108] Ljutić D., Glavina M. (1995). Tubulointerstitial Nephritis with Uveitis Syndrome following Varicella Zoster Reactivation. Nephron.

[109] Mackensen F., Smith J.R., Rosenbaum J.T. (2007). Enhanced recognition, treatment, and prognosis of tubulointerstitial n ephritis and uveitis syndrome. Ophthalmology.

[110] Huang Y., Yang C., Xu X.-F., Xu W., Liu S.-W. (2020). Structural and functional properties of SARS-CoV-2 spike protein: Potential antivirus drug development for COVID-19. Acta Pharmacol. Sin..

[111] Book B.A.J., Schmidt B., Foerster A.M.H. (2021). Bilateral Acute Macular Neuroretinopathy after Vaccination against SARS-CoV-2. JAMA Ophthalmol..

[112] Forchette L., Sebastian W., Liu T. (2021). A Comprehensive Review of COVID-19 Virology, Vaccines, Variants, and Therapeutics. Curr. Med. Sci..

[113] Ichhpujani P., Parmar U.P.S., Duggal S., Kumar S. (2022). COVID-19 Vaccine-Associated Ocular Adverse Effects: An Overview. Vaccines.

[114] WHO Coronavirus (COVID-19) Dashboard WHO Coronavirus (COVID-19) Dashboard with Vaccination Data. https://covid19.who.int/.

[115] Ng X.L., Betzler B.K., Testi I., Ho S.L., Tien M., Ngo W.K., Zierhut M., Chee S.P., Gupta V., Pavesio C.E. (2021). Ocular Adverse Events After COVID-19 Vaccination. Ocul. Immunol. Inflamm..

[116] Eleiwa T.K., Gaier E.D., Haseeb A., ElSheikh R.H., Sallam A.B., Elhusseiny A.M. (2021). Adverse Ocular Events following COVID-19 Vaccination. Inflamm. Res..

[117] Bøhler A.D., Strøm M.E., Sandvig K.U., Moe M.C., Jørstad Ø.K. (2022). Acute macular neuroretinopathy following COVID-19 vaccination. Eye.

[118] Rabinovitch T., Ben-Arie-Weintrob Y., Hareuveni-Blum T., Shaer B., Vishnevskia-Dai V., Shulman S., Newman H., Biadsy M., Masarwa D., Fischer N. (2021). UVEITIS AFTER THE BNT162b2 mRNA VACCINATION AGAINST SARS-CoV-2 INFECTION: A Possible Association. RETINA.

[119] Inagawa S., Onda M., Miyase T., Murase S., Murase H., Mochizuki K., Sakaguchi H. (2022). Multiple evanescent white dot syndrome following vaccination for COVID-19: A case report. Medicine.

[120] Goyal M., Murthy S.I., Annum S. (2021). Bilateral Multifocal Choroiditis following COVID-19 Vaccination. Ocul. Immunol. Inflamm..

[121] Kamoi K., Ohno-Matsui K. (2024). Long Vax in the Eye: Long Post-COVID Vaccination Syndrome Presenting with Frosted Branch Angiitis. Diseases.

[122] Hamad I., Hunter A.C., Szebeni J., Moghimi S.M. (2008). Poly(ethylene glycol)s generate complement activation products in human serum through increased alternative pathway turnover and a MASP-2-dependent process. Mol. Immunol..

[123] Ibrahim M., Ramadan E., Elsadek N.E., Emam S.E., Shimizu T., Ando H., Ishima Y., Elgarhy O.H., Sarhan H.A., Hussein A.K. (2022). Polyethylene glycol (PEG): The nature, immunogenicity, and role in the hypersensitivity of PEGylated products. J. Control. Release.

[124] Frank M.M., Fries L.F. (1991). The role of complement in inflammation and phagocytosis. Immunol. Today.

[125] Noris M., Remuzzi G. (2013). Overview of Complement Activation and Regulation. Semin. Nephrol..

[126] Yang Q., Lai S.K. (2015). Anti-PEG immunity: Emergence, characteristics, and unaddressed questions. Wiley Interdiscip. Rev. Nanomed. Nanobiotechnol..

[127] Hsieh Y.-C., Wang H.-E., Lin W.-W., Roffler S.R., Cheng T.-C., Su Y.-C., Li J.-J., Chen C.-C., Huang C.-H., Chen B.-M. (2018). Pre-existing anti-polyethylene glycol antibody reduces the therapeutic efficacy and pharmacokinetics of PEGylated liposomes. Theranostics.

[128] Ganson N.J., Povsic T.J., Sullenger B.A., Alexander J.H., Zelenkofske S.L., Sailstad J.M., Rusconi C.P., Hershfield M.S. (2016). Pre-existing anti–polyethylene glycol antibody linked to first-exposure allergic reactions to pegnivacogin, a PEGylated RNA aptamer. J. Allergy Clin. Immunol..

[129] Yang Q., Jacobs T.M., McCallen J.D., Moore D.T., Huckaby J.T., Edelstein J.N., Lai S.K. (2016). Analysis of Pre-existing IgG and IgM Antibodies against Polyethylene Glycol (PEG) in the General Population. Anal. Chem..

[130] Elsadek N.E., Lila A.S.A., Emam S.E., Shimizu T., Takata H., Ando H., Ishima Y., Ishida T. (2020). Pegfilgrastim (PEG-G-CSF) induces anti-PEG IgM in a dose dependent manner and causes the accelerated blood clearance (ABC) phenomenon upon repeated administration in mice. Eur. J. Pharm. Biopharm..

[131] Kozma G.T., Mészáros T., Vashegyi I., Fülöp T., Örfi E., Dézsi L., Rosivall L., Bavli Y., Urbanics R., Mollnes T.E. (2019). Pseudo-anaphylaxis to Polyethylene Glycol (PEG)-Coated Liposomes: Roles of Anti-PEG IgM and Complement Activation in a Porcine Model of Human Infusion Reactions. ACS Nano.

[132] Mohamed M., Abu Lila A.S., Shimizu T., Alaaeldin E., Hussein A., Sarhan H.A., Szebeni J., Ishida T. (2019). PEGylated liposomes: Immunological responses. Sci. Technol. Adv. Mater..

[133] Li H., Ice J.A., Lessard C.J., Sivils K.L. (2013). Interferons in Sjögren’s Syndrome: Genes, Mechanisms, and Effects. Front. Immunol..

[134] Du M., Wang X., Mao X., Yang L., Huang K., Zhang F., Wang Y., Luo X., Wang C., Peng J. (2019). Absence of Interferon Regulatory Factor 1 Protects Against Atherosclerosis in Apolipoprotein E-Deficient Mice. Theranostics.

[135] Taylor J.M., Minter M.R., Newman A.G., Zhang M., Adlard P.A., Crack P.J. (2014). Type-1 interferon signaling mediates neuro-inflammatory events in models of Alzheimer’s disease. Neurobiol. Aging.

[136] Schustak J., Twarog M., Wu X., Wu H.Y., Huang Q., Bao Y. (2021). Mechanism of Nucleic Acid Sensing in Retinal Pigment Epithelium (RPE): RIG-I Mediates Type I Interferon Response in Human RPE. J. Immunol. Res..

[137] Pardi N., Hogan M.J., Porter F.W., Weissman D. (2018). mRNA vaccines—A new era in vaccinology. Nat. Rev. Drug Discov..

[138] Shaw A.E., Hughes J., Gu Q., Behdenna A., Singer J.B., Dennis T., Orton R.J., Varela M., Gifford R.J., Wilson S.J. (2017). Fundamental properties of the mammalian innate immune system revealed by multispecies comparison of type I interferon responses. PLoS Biol..

[139] Kerur N., Fukuda S., Banerjee D., Kim Y., Fu D., Apicella I., Varshney A., Yasuma R., Fowler B.J., Baghdasaryan E. (2018). cGAS drives noncanonical-inflammasome activation in age-related macular degeneration. Nat. Med..

[140] Orozco L.D., Chen H.-H., Cox C., Katschke K.J., Arceo R., Espiritu C., Caplazi P., Nghiem S.S., Chen Y.-J., Modrusan Z. (2019). Integration of eQTL and a Single-Cell Atlas in the Human Eye Identifie s Causal Genes for Age-Related Macular Degeneration. Cell Rep..

[141] Dhir A., Dhir S., Borowski L.S., Jimenez L., Teitell M., Rötig A., Crow Y.J., Rice G.I., Duffy D., Tamby C. (2018). Mitochondrial double-stranded RNA triggers antiviral signalling in hum ans. Nature.

[142] Chawla-Sarkar M., Lindner D.J., Liu Y.F., Williams B.R., Sen G.C., Silverman R.H., Borden E.C. (2003). Apoptosis and interferons: Role of interferon-stimulated genes as mediators of apoptosis. Apoptosis.

[143] Li Y., Guo X., Hu C., Du Y., Guo C., Di W., Zhao W., Huang G., Li C., Lu Q. (2018). Type I IFN operates pyroptosis and necroptosis during multidrug-resistant A. baumannii infection. Cell Death Differ..

[144] Stawowczyk M., Van Scoy S., Kumar K.P., Reich N.C. (2011). The interferon stimulated gene 54 promotes apoptosis. J. Biol. Chem..

[145] Thapa R.J., Nogusa S., Chen P., Maki J.L., Lerro A., Andrake M., Rall G.F., Degterev A., Balachandran S. (2013). Interferon-induced RIP1/RIP3-mediated necrosis requires PKR and is lic ensed by FADD and caspases. Proc. Natl. Acad. Sci. USA.

[146] Sarhan J., Liu B.C., Muendlein H.I., Weindel C.G., Smirnova I., Tang A.Y., Ilyukha V., Sorokin M., Buzdin A., Fitzgerald K.A. (2019). Constitutive interferon signaling maintains critical threshold of MLKL expression to license necroptosis. Cell Death Differ..

[147] Fischer S., Gerriets T., Wessels C., Walberer M., Kostin S., Stolz E., Zheleva K., Hocke A., Hippenstiel S., Preissner K.T. (2007). Extracellular RNA mediates endothelial-cell permeability via vascular endothelial growth factor. Blood J. Am. Soc. Hematol..

[148] Kannemeier C., Shibamiya A., Nakazawa F., Trusheim H., Ruppert C., Markart P., Song Y., Tzima E., Kennerknecht E., Niepmann M. (2007). Extracellular RNA constitutes a natural procoagulant cofactor in blood coagulation. Proc. Natl. Acad. Sci. USA.

[149] Prünte C., Flammer J. (1996). Choroidal capillary and venous congestion in central serous chorioretinopathy. Am. J. Ophthalmol..

[150] Iijima H., Iida T., Murayama K., Imai M., Gohdo T. (1999). Plasminogen activator inhibitor 1 in central serous chorioretinopathy. Am. J. Ophthalmol..

[151] Mutter J., Yeter D. (2008). Kawasaki’s disease, acrodynia, and mercury. Curr. Med. Chem..

[152] Stejskal J., Stejskal V.D. (1999). The role of metals in autoimmunity and the link to neuroendocrinology. Neuro Endocrinol. Lett..

